# Automated Fictional Ideation via Knowledge Base Manipulation

**DOI:** 10.1007/s12559-015-9366-4

**Published:** 2016-01-11

**Authors:** Maria Teresa Llano, Simon Colton, Rose Hepworth, Jeremy Gow

**Affiliations:** Department of Computing, Goldsmiths, University of London, London, UK

**Keywords:** Fictional ideation, Computational creativity, Knowledge bases

## Abstract

The invention of fictional ideas (ideation) is often a central process in the creative production of artefacts such as poems, music and paintings, but has barely been studied in the computational creativity community. We present here a general approach to automated fictional ideation that works by manipulating facts specified in knowledge bases. More specifically, we specify a number of constructions which, by altering and combining facts from a knowledge base, result in the generation of fictions. Moreover, we present an instantiation of these constructions through the use of ConceptNet, a database of common sense knowledge. In order to evaluate the success of these constructions, we present a curation analysis that calculates the proportion of ideas which pass a typicality judgement. We further evaluate the output of this approach through a crowd-sourcing experiment in which participants were asked to rank ideas. We found a positive correlation between the participant’s rankings and a chaining inference technique that automatically assesses the value of the fictions generated through our approach. We believe that these results show that this approach constitutes a firm basis for automated fictional ideation with evaluative capacity.

## Introduction

Ideation is a portmanteau word used to describe the process of generating a novel idea of value. Fictional ideation therefore describes the production of ideas which are not meant to represent or describe a current truth about the world, but rather something that is in part, or entirely, imaginary. As such, its purposes include unearthing new truths and serving as the basis for cultural creations like stories, advertisements, poems, paintings, games and other artefacts.

A major field of study within computational creativity research involves designing software that exhibits behaviours perceived as creative by unbiased observers [3]. As an example, *The Painting Fool*^1^ system [2], an automated artist, has produced pieces which have been exhibited in real and online galleries. Similarly, the work by Colton et al. [4] reports on the automatic generation of poems, where the poem represents a response to articles from the Guardian newspaper. In both these cases, as in the majority of the systems developed so far within computational creativity research, there is no idea generation undertaken explicitly. In many projects, especially applications to natural language generation such as neologism production [38], which are communicative in nature, it is entirely possible to extract ideas from the artefacts produced. However, it is fair to say that the software used in these projects is not performing ideation in order to produce artefacts; they are rather producing artefacts which enable the reader to interpret them via new ideas.

In the creative arts and the creative industries, the production of fictional ideas around which to write stories, paint pictures or design advertisements, is an essential activity. With this in mind, the work presented here, which is part of the WHIM^2^ project (an acronym for the *What-if Machine*), aims to undertake the first large-scale study of how software can invent, evaluate and express fictional ideas.

It is important to highlight that we are taking an engineering approach to fictional ideation, so our aim is to build a working computational system able to generate textual what-if ideas as a study in computational creativity. It is also beyond the scope of this paper to add to the discourse surrounding the nature of fictional ideas. However, we contextualise what this concept means within our work. For the purpose of this paper, a fictional idea is a piece of text which describes a scenario that an unbiased observer would be likely to deem as imaginary. We acknowledge that fictionality also exists in scenarios that would be deemed ordinary, but don’t exist given the uniqueness of the scenario. So, for instance, a detailed characterisation such as a man called Brian who has long curly hair living in Bristol with a woman called Maria, is fictional (to the best of our knowledge) in the sense that such a scenario doesn’t actually exist, but not fictional in the sense that such a scenario couldn’t exist. If Brian had 17 arms, however, this would be fictional in both senses, and it is the latter sense—where a scenario is unlikely—that we are pursuing with the What-if Machine project. We discuss this further in the paper.

Automatically generating interesting fictional ideas is a challenging task. An idea which makes sense as a fiction is not necessarily one which excites the mind. For instance, the idea: “What if there was a chair with five legs?” is coherent, it has saliency and is largely fictional, given that most chairs have three or four legs only. However, it takes some work to imagine a scenario in which a five-legged chair would be of particular interest. Hence, this idea is unlikely to enthuse people to play around with it in their mind by dreaming up humorous, dangerous or ridiculous scenarios in which the idea features. A good fictional idea distorts the world around it in useful ways, and these distortions can be exploited to spark new ideas, to interrogate consequences and to tell stories. To illustrate these points, the ideas below represent one-line summaries of the plots of two well-known stories:

What if we could give life to a being created by combining the body parts of dead people?

What if there are other worlds, running parallel to ours, which can only be accessed by children?

We can describe such ideas as being rich in narrative potential (NP). That is, they might provoke someone exposed to them to imagine plot lines, characters, dialogues and other narrative elements which somehow involve the key concepts in the idea. However, it is important to note that audience appreciation of the value of an idea is often relative to the way in which the idea is presented and the context in which this presentation occurs.

In this paper, we first present an account of what we mean by fictional ideation in the context of the WHIM project. Based on this definition, we present an approach to fictional ideation which manipulates information from knowledge bases (KBs). Largely, the approach consists of altering facts from a KB in order to produce a fiction and combining these with facts so as to produce fictional scenarios with NP. The different combinations for the generation of fictional ideas explored here are heuristic in nature and constitute only a set of possible transformations that can be carried out in order to obtain fictional ideas; therefore, our work is by no means an exhaustive list of all the transformations that can be achieved but rather different types that have been identified and used within the WHIM project so far. However, we show throughout the paper that our approach to fictional ideation based on KB manipulation is successful at producing fictional ideas of different types.

Because of the heuristic nature of our approach, we have conducted a *curation analysis* of it applied over ConceptNet,^3^ a semantic network of common sense knowledge produced by sophisticated Web mining techniques at the MIT Media Lab [17]. The analysis consists of curating the output by selecting the proportion of ideas which are typical in the sense of being both understandable and largely fictional. This analysis provides a baseline evaluation and a first measure of progress within the approach. Additionally, we present the results from a crowd-sourcing exercise involving 135 participants, where people were exposed to ideas in a controlled way, with the aim of evaluating components of ideas that could be used to predict overall value. A central hypothesis of the WHIM project is that the NP of an idea can be estimated automatically and used as a reliable estimate of the idea’s worth. Hence, the crowd-sourcing study had NP as a focal point, and we tested an automated approach which estimates whether an idea has much NP, or little. As discussed below, we found that, in general, people ranked those ideas that were assessed as having much potential higher than those assessed as having little. We present further statistical analysis of the results, which enables us to conclude by describing future directions for the WHIM project.

This paper is an extended version of the work presented in the Computational Creativity Workshop collocated with AISB 50 [19] and the 5th International Conference on Computational Creativity [21]. In [19], a single transformation technique was proposed by negating relations from ConceptNet facts. The approach was evaluated through a pilot study in which 10 participants ranked a list of fictional ideas, while in [21], we presented a crowd-sourcing experiment with 135 participants who ranked different types of fictional ideas and performed a statistical analysis of the results. Here, we significantly extended this work by specifying a number of general constructions, applicable to KBs of common sense knowledge, which generate different types of fictions. Moreover, here we formalised these constructions through the use of first-order predicate logic (FOPL), which is an approach widely used in natural language processing. We also report a more exhaustive evaluation of our approach through a curation analysis that provides an initial estimation of the value of the automatically generated output. In this extended version, we also provide further insights into what fictional ideation means in the context of the WHIM project, as well as a more complete account of related work. Finally, a first prototype of the system has been completed and is available online^4^ with different types of ideas being generated, some of which use implementations of the work presented here.

## Background and Related Work

In the majority of the generative systems developed so far within computational creativity research, there is no idea generation undertaken explicitly. However, there are some exceptions to this. For instance, Pereira [29] implemented a system based on the psychological theory of conceptual blending put forward by Fauconnier and Turner [6]. By blending two theories about different subject material, novel concepts which exist in neither domain emerge from the approach. Using blending to reason about such fictional ideas was harnessed for various creative purposes, including natural language generation [31], sound design [24], and the invention of character models for video games [30]. Similarly, the ISAAC system, developed by Moorman and Ram [26], implements a theory for creative understanding based on the use of an ontology to represent the dimensions of concepts. By altering the dimensions of existing concepts within the ontology, for instance considering a temporal object, e.g. the concept of *days*, as a physical one, the system is able to create novel concepts such as *days that fly*.

In addition, the work in [7] shows the use of creative analogies in which problems of environmental sustainability are addressed by creating designs inspired by the way things work in nature. For instance, birds’ beaks inspired the design of trains with noise reduction. Although ideation in this approach is being used for inspiration and not to create literal representations, this work shows the potential of using creative analogies for fictional ideation, as is the case of the Copycat system [11], by Hofstadter. The basic principle of this approach is that one can achieve similar outputs by identifying analogies from previously seen examples and then “copying” generation mechanisms so as to achieve a similar output. More specifically, this approach seeks to solve problems such as “abc is to abd as ijk is to what?”

To achieve the process mentioned above, Hofstadter follows a technique called *slipping* [10]. The reasoning behind this originated on the analysis of counterfactuals, which represent variants of situations that have happened in real life. These variants are features of such situations that we let “slip” from our minds, while the other features remain the same. Depending on the context of the situation, we let slip some features more easily than others. In general, slipping considers that objects, events, actions, etc. are composed of some tight and some loose elements that differ according to the context, in which the loose elements are more easily replaced. The Copycat system uses this technique by slipping properties from one concept to another. That is, when two concepts are closely related, one concept may slip into another. Some of the constructions to fictional ideas proposed here also follow this technique. That is, based on an initial transformation of a fact, the system searches for concepts whose properties intercept with the concepts in the transformation and selects which of them are suitable to form interesting consequences. This ensures that the different elements of the fictional idea are connected and are somehow consistent with the initial transformation. However, contrary to counterfactual reasoning (which slipping is based on), the link between the transformation and the consequence is also fictional in the sense that it did not initially backed or preceded the transformed fact. Furthermore, our approach is flexible in the sense that it explores different levels of fictionality by slipping loose and tight features of facts. As future work, we will study the appreciation of this levels of fictionality through a measure of plausibility which we hope to correlate with further crowd-sourcing studies.

The creative generation of characters for stories has also been explored in the context of fictional ideation. Examples of this are the Party Quirks [22] and the Flux Capacitor [39] systems. The former is a digital improvisational theatre game that allows the generation of imaginary characters by manipulating their stereotypical attributes, e.g. a clumsy ninja. The Flux Capacitor, on the other hand, defines conceptual start and end points to transform the description of characters within a narrative, e.g. from good to evil, from rich to poor. These characters are computationally modelled as dynamic blends; that is, they can be used as the input for story generators and developed throughout a narrative. The generation of fictional objects that can play functional roles in stories has also been studied by Li and Riedl [16]. This is achieved by using partial-order planning and analogy to find relations between typical properties and events of different objects, giving rise to new concepts such as a phone that can transmit the flu.

Most of these approaches have in common what Steven Johnson calls “the adjacent possible” in his book *Where Good Ideas Come From: a Natural History of Innovation* [13]. This principle specifies that the best ideas are those that are close or adjacent to existing concepts. This is in line with the findings of Wundt [40], who points out that the hedonistic value of an artefact increases with novelty in the first instance, but then decreases as the novelty further increases, as it becomes more difficult to place the artefact into a context. Our findings through the evaluation carried out here indicate that this is also true of fictional ideas. As a result, we use analogy at the level of KBs in order to identify similarities between the properties of concepts. We further strengthen the matches through the use of contextual semantic similarity tools, such as Disco [15], which use vector space models for identifying similarities between the vector representations of two terms. These models allow us to favour matches such as $\textit{running} \mapsto \textit{riding a horse}$ over matches such as $\textit{running} \mapsto \textit{learning}$—such matches are identified by the first step of analogy, i.e. properties comparison through facts from the KB.

Overall, our approach to automated fictional ideation presents and ranks fictional ideas according to a measure of NP. In order to illustrate its capabilities, we use ConceptNet, a KB whose mined knowledge is represented as facts. These facts comprise relations between concepts that are expressed as words and short phrases, in a network-like structure. There are many relations, including:

**Table:** 

*Antonym, AtLocation, CapableOf, Causes, CreatedBy, Desires, HasA, HasProperty, IsA, InstanceOf, LocatedNear, MadeOf, MemberOf, NotHasA, NotIsa, PartOf, SimilarTo, Synonym, UsedFor*	

Each fact is given a score from 0.5 upwards, which estimates the likelihood of the relation being true based on the amount of evidence mined. We extracted the bare information from ConceptNet into a set of tuples of the form:

[LHSConcept,Relation,RHSConcept,Score].

As examples, the following are facts in ConceptNet about particular animals: [camel, IsA, animal, 7.0], [bee, CapableOf, make_honey, 2.0], [cat, Desires, play_with_string, 6.0], etc. Some relations are included in many facts, while others are included in far fewer.

Liu and Singh [17] describe various uses for ConceptNet, including finding contexts around a concept, making analogies and constructing chains of inference. The latter of these is of interest here. Liu and Singh provide an example of such a chain:

ConceptNet can generate all the temporal chains between “buy food” and “fall asleep”. One chain may be: “buy food” $\rightarrow$ “have food” $\rightarrow$ “eat food” $\rightarrow$ “feel full” $\rightarrow$ “feel sleepy” $\rightarrow$ “fall asleep”. Each of these chains can be seen as being akin to a “script”. $\ldots$ By knowing that “buy steak” is a special case of “buy food”, $\ldots$ we can now make the inference “fall asleep”.

An inference chaining approach has been used in the Emotus Ponens system, by Liu et al. [18], for affective text classification. As described below, we similarly employ such chains to estimate the NP of fictional ideas.

As an implementation infrastructure, we have used FloWr [1], a framework for implementing creative systems as scripts over processes that can be manipulated visually as flowcharts. Providing details of how this system works is beyond the scope of this paper. However, we give the details of the individual flowchart nodes we have employed in order to present our approach.

Next, we describe the concept of fictional ideation and the value of idea-driven fiction as a mechanism for the generation of creative artefacts. Then, we specify our approach, followed by the curation analysis and the results from the crowd-sourcing experiment. We conclude by describing some future developments for automated fictional ideation.

## Fictional Ideation

Thomas Reid’s *Essays on the Intellectual Powers of Man* [32] sought to trace the history of the term idea in seventeenth and eighteenth century philosophy and is an early contribution to ongoing attempts to define and understand what we mean by the term idea in the context of human knowledge and understanding.

A persistent theme in much of this work has been the contested question of where ideas come from. While some theorists have proposed that ideas exist as knowledge independent from, but accessible to, individuals, others argue that ideas originate from an individual’s experiences and perceptions of the world around them. More recently, ideas and concepts (the terms are sometimes used synonymously) are understood as being the result of either an individual’s association of a new object with one it resembles, or an individual placing objects in a specific category according to the characteristics they are perceived to have.

In the *Big book of concepts* [27], Murphy tries to unify some of these differences by arguing that understanding human thinking requires an approach that combines of all these theories, and that an external general knowledge is drawn upon in combination with personal experience in the formation of ideas and concepts. As such, Murphy’s work would be a good starting point for readers wishing to explore the ways that computational creativity and ideation might contribute to thinking about concepts and ideas. We note the theoretical history of the term idea here only insofar as it illustrates the need for a degree of precision in the way we use the terms idea, fiction and fictional ideation.

In this paper, and in the WHIM project itself, we understand ideas to refer to modifications of knowledge in which the perceptions we hold about existing concepts of the world are altered and new representations are produced. That is, an idea modifies the ontological status of current concepts by manipulating their attributes as well as their relationships with other concepts, resulting in representations that do not necessarily correspond to any physical or abstract object in the world. In this way, the concept of *a dog that is able to jump* is not considered to be an idea because it is a concept with which we already have familiarity. However, the concept of *a dog that knows how to read* is (for most) unfamiliar and results in a modification of the known relation between the concepts dog and reading. Though our example of a literate dog may well be fictional, it is important to note that our definition of idea as it is presented here does not presuppose fictionality. Nevertheless, the WHIM project is specifically concerned with ideas whose plausibility might require us to suspend our disbelief, that is fictional ideas.

Although most of us have an intuitive sense of what we mean by *fiction*, we have found that distinctions between the factual and the fictional blur when either is subjected to interrogation. Indeed, the term fiction is difficult to define. Several theoretical approaches and the problems they present are examined by Schaeffer in the Living Handbook of Narratology (LHN) [36]. A useful working definition taken from the LHN might be: “a representation portraying an imaginary/invented universe or world” [36, Paragraph 9]. This definition reinforces an approach whereby fiction is defined against a factual (or at least a non-imaginary/non-invented universe or world) and relies upon an assumption that factual narrative is referential, whereas fictional narrative has no reference (at least not in “our” world). This is useful in that it begins to demarcate ideas that are fictional from other kinds of ideas produced in a creative fashion. The What-if Machine is a fictional ideation system, and so it is this term we use here to describe *what-if* ideas rather than terms such as *novel* which, otherwise, might have been more appropriate.

It is important to consider degrees of fictionality in determining the value of what-if style ideas. What-ifs are not fully developed narratives. Rather, they are short expressions of a fictional idea and can be described as mini-narratives. With this in mind, imagine that Virginia Woolf’s *Mrs Dalloway* had been developed from the what-If idea: “What if there was a woman who spent a day preparing to host a party at which she heard about the suicide of a man?” Although Mrs Dalloway is a work of fiction (to whatever extent it is influenced by the author’s experiences), the question does not have a high fictionality value. That is because the world we understand to be real, would not have to change in any significant way in order for this proposed occurrence to actually happen.

By contrast, imagine that the following what-if idea was a starting point for Mary Shelley’s *Frankenstein*: “What if we could give life to a monster created by combining the body parts of dead people?” Although both novels are works of fiction, when presented as what-if ideas, the latter example has a higher fictionality. One measure of fiction is therefore how far one is taken from the “real” world by the imagined world. A further level of fiction can be found in the idea: “What if a zombie rugby-tackled a ghost and broke his leg?” For our current purposes, this is a level of fiction too far. This is not a fiction about the world we understand to be real; rather it is a fiction about a world we already know to be fictional: one where zombies and ghosts (co)exist. As such, it is not enough that a what-if idea represents an “imaginary/invented universe or world”, it has to take us there from the familiar territory of our own world.

Clarifying the parameters of what we mean by fictionality (as far as it is possible to do so) is important because we need to be able to measure the What-if Machine’s ability to produce fictional ideas. Exploring levels of fictionality is part of our future work; indeed, the software itself will need an ability to assess such levels. In view of this, we might amend the working definition of fiction above in order to provide an account of what constitutes a successful what-if idea: a good fictional what-if idea is one that presents a character, event or scenario that transforms or distorts the “real” world in the imagination of the reader without requiring him or her to leave it entirely.

### Idea-Driven Fiction

In the WHIM project, we are specifically tasked with producing software capable of fictional ideation, and therefore fictional elements must be apparent in the short “mini-narratives” presented in these what-if ideas. As such, they often present scenarios that probably wouldn’t or couldn’t happen in the world we know. As consumers of narrative, our pleasure is often, in large part, the result of an artist or writer’s ability to successfully immerse us in a world utterly different to our own and convince us to suspend our disbelief enough to invest in that world and the characters that inhabit it. However, we also recognise that the degree of plausibility of an individual scenario does not necessarily make for a more successful story, poem or painting. Indeed, many critically acclaimed works of art, across all media, represent worlds that closely resemble our own. Our pleasure in these works tends to be derived from other elements: the psychology of their characters, for example, or their exploration of a particular theme.

In view of this, we aim to produce a What-if Machine capable of generating ideas associated with different dimensions of fictionality. Currently, through the Flux Capacitor system [39]—developed by Tony Veale as part of the WHIM project—fictional ideas about interesting character transformations are generated by the What-if Machine, e.g. “What if strong athletes were to lose their fans, bow down to kings and become powerless serfs?” Also developed by Veale, the system produces ideas about utopias and dystopias, and the consequences that they bring, e.g. “What if the world suddenly had lots more gods? Then there would be more beasts, since gods create the monsters that live in the lairs that protect beasts.” In [20], we have also explored how the What-if Machine can be used for the generation of fictional ideas that can be employed in developing various aspects of video games, such as game mechanics, ending conditions (when a player loses or the games finishes), locations, objects. Currently, we are also working on the generation of ideas suitable for advertising and musical theatre using the approach presented in this paper. Through these different domains, we are able to explore degrees of fictionality (as they relate to plausibility) as well as dimensions of fictionality (elements of fictional worlds), which are both of interest within the WHIM project.

We believe the applications for this type of system are broad. As an autonomous agent, we envisage the What-if Machine would be able to create and evaluate material with minimal input, as well as contribute to the creative process, whether at the level of an inspirational system, a tool or a collaborator. Furthermore, we believe the What-if Machine could be used to adjust a scenario “on-the-fly” with invented ideas. This would be particularly useful for settings such as that of video games and creative writing.

## Methodology

Based on our definition of what is considered a good fictional idea within the What-if Machine context, our approach consists of applying controlled alterations and combinations of facts, such that the produced ideas are fictional but within a frame of reality understandable by the user. Common sense KBs are therefore a very good source of information in order to achieve this purpose, as they store information about the world in the form of facts which specify relations between concepts. Different KBs, such as ConceptNet, Reverb [5], contain various details about the information they store, such as the Web source, frequency the fact has been seen. However, there are three intrinsic elements associated with this knowledge, and common to most KBs, which are of interest for the work presented here, namely concepts *C*, relations *R* and facts *F*. A fact relates two concepts through a relation in a tuple of the form:

$$\langle \textit{c}_{1},\textit{r},\textit{c}_{2} \rangle \in \textit{F}$$

where $\textit{c}_{1}\in \textit{C}$ and represents the left-hand side concept, $\textit{c}_{2}\in \textit{C}$ and represents the right-hand side concept, and $\textit{r}\in \textit{R}$ and represents the relation that associates the left- and right-hand side concepts.

The What-if Machine is therefore tasked to manipulate real-world knowledge in order to generate fictions. Assuming a closed-world representation from a KB, a fiction is generated by manipulating the elements of a fact in a way in which the resulting tuple is not part of the KB. Therefore, we define a *fiction* as a tuple $\tau = \langle \textit{c}_{1},\textit{r},\textit{c}_{2} \rangle$ such that $\tau \notin \textit{F}$. For instance, assuming ConceptNet as our closed-world representation, $\langle \textit{cat, Desires, milk} \rangle$ is a fact in ConceptNet; however, the fact $\langle \textit{cat, Desires, bone} \rangle$ does not appear in ConceptNet; therefore, we say this tuple represents a fiction.

Note that the tuples resulting from this manipulation of knowledge from a KB may or may not be fictional with respect to the real world. That is because KBs do not contain all the information about the world; however, they are fictional with respect to the KB since we assume a closed-world representation.

### Generating Fictions

There are two basic transformations that can be carried out in order to manipulate facts from a KB and which achieve conceptual changes that lead to the generation of fictions: (1) altering the relation between two concepts and (2) altering the concepts involved in a fact. We specify particular examples of these transformations next.

*Transformation 1* Altering the relations between two concepts that are already related is a common mechanism used in fiction. This is usually achieved by enabling properties that cannot normally occur, amplifying or reducing current skills or functions, disabling properties, etc. There are three requirements to apply this type of transformation: (1) the new relation should be different from the current relation, (2) the new relation should be a suitable replacement based on the involved concepts, and (3) the resulting relationship should not already occur in the real world. This is specified in formula (1):

1 $$\begin{aligned}&\textit{alter} \textit{Relation}(\langle \textit{x,r,y} \rangle) \\ & \quad = \{\langle \textit{x,l,y} \rangle \,\vert \, \textit{l} \ne \textit{r} \,\wedge \, \textit{validPOS(x,l,y)} \\ & \qquad \wedge \, \langle \textit{x,l,y} \rangle \notin \textit{F} \} \end{aligned}$$

where *validPOS*(*x,l,y*) specifies if the concepts *x* and *y* correspond to the right part of speech (POS) associated with relation *l*. For instance, *validPOS*(*dog, CapableOf, high*) *= false* since the concept “high” is an adjective. On the contrary, *validPOS*(*dog, CapableOf, jump*) *= true* since both concepts have the right POS; that is, “dog” is a noun and “jump” is a verb. Examples of this transformation are:

$$\begin{aligned}&\textit{alterRelation}(\langle \textit{bird, CapableOf, fly}\_\textit{in}\_\textit{air}\rangle) \\ & \quad = \{\langle \text {bird, NotCapableOf, fly}\_\text {in}\_\text {air}\rangle, \langle \text {bird, UsedFor, fly}\_\text {in}\_\text {air} \rangle, \\ & \qquad \langle \text {bird, AfraidOf, fly}\_\text {in}\_\text {air} \rangle, \ldots \} \end{aligned}$$

*Transformation 2* Techniques such as anthropomorphization (also called personification) or zoomorphication, in which human properties are attributed to animals or things, or vice versa, are very common literary devices used in storytelling and other kinds of arts. This type of conceptual change can also be achieved by manipulating the concepts involved in the facts within a KB. Similar conditions to the previous transformation are required, as specified in formula (2):

2 $$\begin{aligned} &\textit{alter}\textit{Concept}(\langle \textit{x,r,y} \rangle) \\& \quad = \{\langle \textit{x}^{\prime }\textit{,r,y}^{\prime } \rangle \,\vert \, (\textit{x}^{\prime } \ne \textit{x} \,\vee \, \textit{y}^{\prime } \ne \textit{y}) \\ & \qquad \wedge \, \textit{validPOS(x}^{\prime }\textit{,r,y}^{\prime }\textit{)} \, \wedge \, \langle \textit{x}^{\prime }\textit{,r,y}^{\prime } \rangle \notin \textit{F} \} \end{aligned}$$

where either one or both concepts of an input fact are altered. The alternative concept(s) must correspond to the right POS according to the relation associated with the input fact, and the resulting relationship must not appear already in the KB. To illustrate, some example fictions produced through this transformation are:

$$\begin{aligned} &\textit{alterConcept}(\langle \textit{horse, LocatedNear, stable}\rangle) \\ & \quad = \{\langle \text {dolphin, LocatedNear, stable} \rangle, \langle \text {horse, LocatedNear, space} \rangle, \\ & \qquad \langle \text {dolphin, LocatedNear, space} \rangle, \ldots \} \end{aligned}$$

However, applying these basic transformations without any kind of control would yield fictions which may be nonsensical, difficult to interpret or simply not interesting. For instance, among the possible fictions generated by applying transformation 1 to the fact $\langle$*dog*, *desires*, *bone*$\rangle$ there are:

- $\langle$dog, likes, bone$\rangle$
- $\langle$dog, partOf, bone$\rangle$
- $\langle$dog, afraidOf, bone$\rangle$

Fiction (a) is not interesting since it does not alter the original fact significantly so as to change the world around it, while fiction (b) cannot be easily interpreted.^5^ However, we can say with confidence that fiction (c) is more interesting, since it inverts the relation expressed in the fact, converting a desire into a fear, has sense and can be easily interpreted by a user.

We have explored different *constructions* which combine facts and fictions into interesting fictional ideas. In the next section, we will present some general constructions explored in the WHIM project.

## General Constructions for Fictional Ideation

The transformations presented above represent basic manipulations that can be performed on facts of a KB in order to obtain suitable alterations of reality. However, fictional ideas are not only the result of altering the ontological status of individual facts but also they result from modifying more complex structures of interconnected facts. These more complex alterations of reality use the basic transformations presented above in order to combine facts and fictions in different ways; we call these methods of fictional ideation *constructions*. We have investigated various constructions that produce such fictional ideas. The result from each construction is a set of tuples, whose elements are combinations of facts and fictions. These tuples can be interpreted or rendered in different ways. We have carried out some experiments using ConceptNet and the FloWr flowcharting system and have used different renderings to present the output. Details about these experiments are provided alongside the specification of each construction.

*Construction 1. Altering the nature of a relation* One of the most straightforward ways of controlling the generation of fictions is by modifying the nature of the relations expressed by facts. This includes inversions such as *What if people could fly?*, arising from the fact “people can’t fly”, or making the relation stronger, e.g. “people enjoy jumping” becomes *What if people were addicted to jumping?*.

To achieve these types of alterations, facts are transformed by replacing their relation for a conceptually related alternative, through either a synonym or antonym connection. For instance, the words *able* and *unable* are conceptually related to the relation *capable*; however, the word *use* is not. Furthermore, the original relation and the alternative should not be too semantically close. To illustrate, the conceptual similarity between the concepts capable and able is 0.707, while the conceptual similarity between capable and unable is 0.265—the concept similarity values were obtained through the UMBC phrase similarity Web service^6^—therefore, our hypothesis is that selecting *unable* as the replacement for the relation would yield more interesting fictions. This construction is specified in formula (3):

3 $$\begin{aligned}&\textit{alterRelationNature}(\langle \textit{x,r,y} \rangle) \\&\quad =\{\langle \textit{x,l,y} \rangle \,\vert \, \langle \textit{x,l,y} \rangle \in \textit{alterRelation(x,r,y)} \,\wedge \\&\quad \textit{l} \in \textit{conceptuallyRelated(r)} \,\wedge \, \textit{notCloselySimilar(l,r)} \} \end{aligned}$$

where *conceptuallyRelated*(*r*) returns a set of words which are related to *r* based on concept similarity, and *notCloselySimilar*(*l,r*) specifies if the similarity between *l* and *r* is between an upper threshold of 0.7 and lower threshold of 0.1—these thresholds have been selected through experimentation, where words outside the range are discarded because they are semantically too close or too far from the original word, producing uninteresting fictions.

**Fig. 1:**
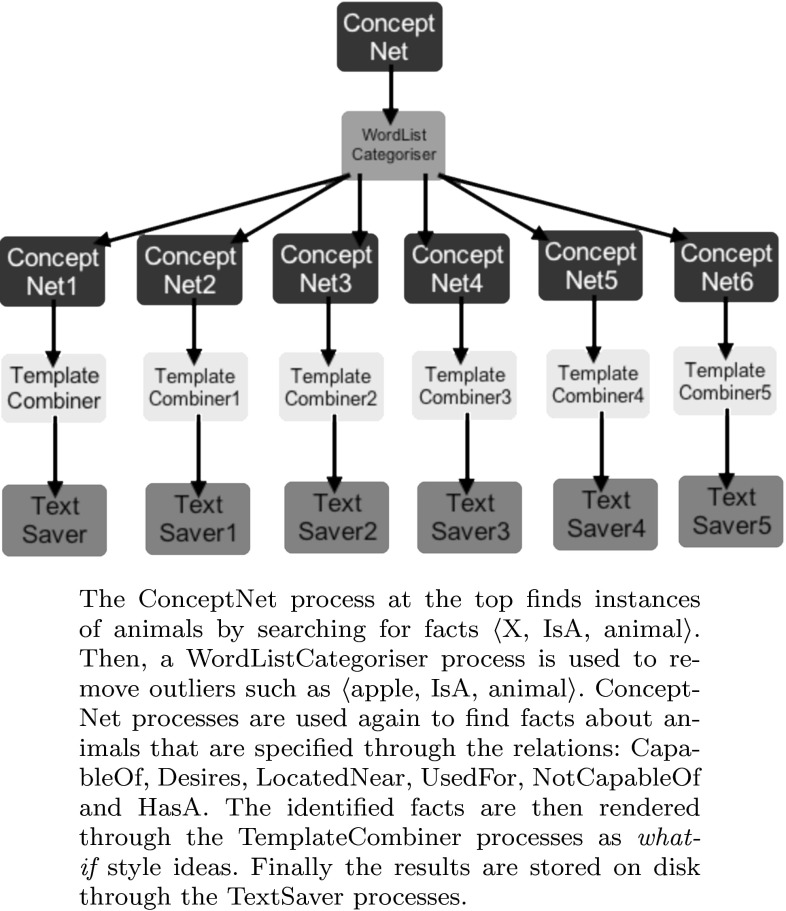
Flowchart used to generate fictional characters in the context of Disney films

We applied this construction to generate fictions about Disney characters. Figure 1 shows the flowchart used to achieve this. For instance, the fact $\langle$Cat, Desires, Milk$\rangle$ is rendered as “What if there was a little cat who was afraid of milk?”—where the change from *Desires* to *AfraidOf* has been made following formula (3).

*Construction 2. Assigning a new type* Using instances of concepts as new types for other concepts is an effective mechanism used to produce fictions. An example of this is the well-known tale of the prince that becomes a frog, or stories from films in which a beggar becomes a rich banker, or a child that suddenly becomes an adult, etc. The details of this construction are given in formula (4):

4 $$\begin{aligned}&\textit{assign}\textit{Type}(\langle \textit{x,is}\_\textit{a,t}_{1} \rangle) \\&\quad =\{(\langle \textit{x,is}\_\textit{a,t}_{1} \rangle, \langle \textit{x,is}\_\textit{a,y} \rangle) \,\vert \, \langle \textit{y,is}\_\textit{a,t}_{2} \rangle \\&\qquad \in \textit{F} \,\wedge \textit{t}_{1} \ne \textit{t}_{2} \,\wedge \, \langle \textit{x,is}\_\textit{a,y} \rangle \\&\qquad \in \textit{alterConcept}(\langle \textit{x,is}\_\textit{a,t}_{1} \rangle) \} \end{aligned}$$

Observe that the construction is guided through the “is_a” relation, which specifies that the type of the left-hand side concept is the concept in the right-hand side. This relation commonly appears in all KBs, most probably with a different name, but with the same semantic meaning. The result of this construction is a set of pairs of tuples, which specify the original type and the new type of concept *x*.

**Fig. 2:**
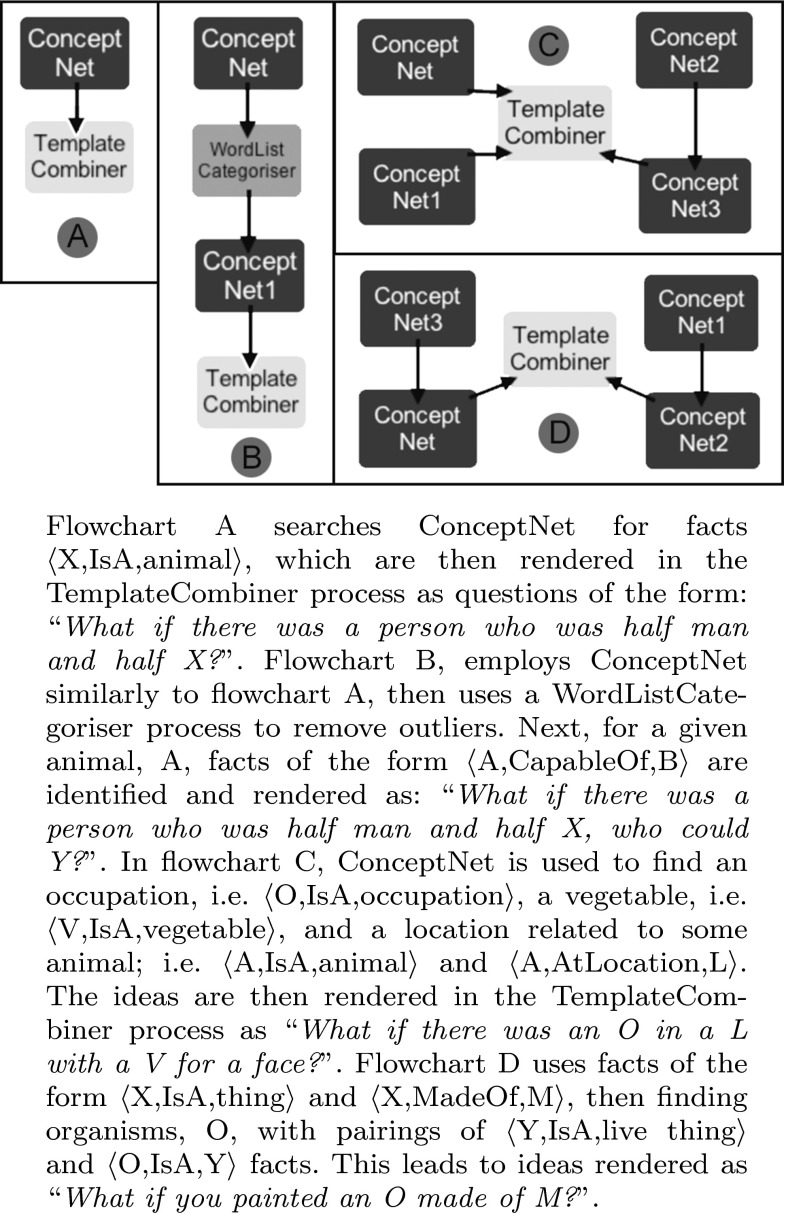
Ideation flowcharts using ConceptNet

We experimented with this construction via flowchart A in Fig. 2. In particular, working in a story-generation context, we took inspiration from the opening line of Franz Kafka’s 1915 novella *The Metamorphosis*:

One morning, as Gregor Samsa was waking up from anxious dreams, he discovered that in his bed he had been changed into a monstrous verminous bug.

Flowchart A finds instances of animals by searching ConceptNet for facts $\langle$X, IsA, animal$\rangle$. These are then rendered in the *TemplateCombiner* process as questions of the form: “What if there was a person who was half man and half X?”

*Construction 3. Assign a property* Another controlled way of producing fictions is to assign new properties to concepts. This can be guided once a new type has been attributed to a concept. For instance, if we assign the type “bird” to the concept “man”, we can assume that all the properties associated with birds can now be associated with men. For example, the property of birds being able to fly becomes *What if there was a man who could fly?* This is specified in formula (5):

5 $$\begin{aligned}&\textit{assign}\textit{Property}(\langle \textit{x,is}\_\textit{a,t}_{1} \rangle) \\&\quad =\{(\langle \textit{x,is}\_\textit{a,t}_{1} \rangle, \langle \textit{x,is}\_\textit{a,y} \rangle, \langle \textit{x,r,p} \rangle) \,\vert \\&\quad (\langle \textit{x,is}\_\textit{a,t}_{1} \rangle, \langle \textit{x,is}\_\textit{a,y} \rangle) \in \textit{assignType(}\langle \textit{x,is}\_\textit{a,t}_{1} \rangle \textit{)} \\&\quad \wedge \, \langle \textit{y,r,p} \rangle \in \textit{F} \,\wedge \, \langle \textit{x,r,p} \rangle \notin \textit{F} \} \end{aligned}$$

Flowchart B in Fig. 2 employs ConceptNet similarly to flowchart A in the previous example and then uses a *WordListCategoriser* process to remove outliers such as $\langle$my_husband,IsA,animal$\rangle$. Then, for a given animal, A, facts of the form $\langle$A,CapableOf,B$\rangle$ are identified and rendered as: “What if there was a person who was half man and half X, who could Y?”

Flowchart D of Fig. 2 provides another example of this type of fiction generation. Here, we produced ideas for paintings by finding materials, M, using facts of the form $\langle$X,IsA,thing$\rangle$ and $\langle$X,MadeOf,M$\rangle$, then finding organisms, O, with pairings of $\langle$X,IsA,live_thing$\rangle$ and $\langle$O,IsA,X$\rangle$ facts. This led to ideas such as painting a dolphin made of gold, a reptile made of wood and a flower made out of cotton.

*Construction 4. Alter assigned property* Similar to the previous construction, here fictions are achieved by assigning a new property to a concept; however, this property is then altered, as specified in construction 1, so as to produce a twist in the generated idea. The “prince that becomes a frog but can speak” is an example of this type of fiction, which follows from the altered property that frogs cannot speak. Details of this construction are given in formula (6):

6 $$\begin{aligned}&\textit{alterAssignedProperty}(\langle \textit{x,is}\_\textit{a,t}_{1} \rangle) \\&\quad =\{(\langle \textit{x,is}\_\textit{a,t}_{1} \rangle, \langle \textit{x,is}\_\textit{a,y} \rangle, \langle \textit{x,l,p} \rangle) \,\vert \\&\quad (\langle \textit{x,is}\_\textit{a,t}_{1} \rangle, \langle \textit{x,is}\_\textit{a,y} \rangle) \in \textit{assignType(}\langle \textit{x,is}\_\textit{a,t}_{1} \rangle \textit{)} \\&\quad \wedge \, \langle \textit{y,r,p} \rangle \in \textit{F} \,\wedge \, \langle \textit{y,l,p} \rangle \in \textit{alterRelationNature(y,r,p)} \} \end{aligned}$$

Switching the CapableOf (CO) relation to NotCapableOf in Flowchart B, and then altering it, enabled us to produce ideas suggesting a person who became an animal, but retained some human qualities.

*Construction 5. Intersecting types* The point of this construction is to combine outputs from constructions 3 and 4, so as to enrich the fictions about a concept, by combining different fictions of them. Imagine for instance a “prince that becomes a frog, and can speak, but cannot jump”. We specify this construction in formula (7):

7 $$\begin{aligned} \textit{intersect}(&\{\langle \textit{x,is}\_\textit{a,t}_{1} \rangle, \langle \textit{x,is}\_\textit{a,y} \rangle, \langle \textit{x},\textit{r}_{1},\textit{p}_{1} \rangle, \ldots, \langle \textit{x},\textit{r}_{n},\textit{p}_{n} \rangle \}) \\&= \{(\langle \textit{x,is}\_\textit{a,t}_{1} \rangle, \langle \textit{x,is}\_\textit{a,y} \rangle, \langle \textit{x},\textit{r}_{1},\textit{p}_{1} \rangle, \ldots, \\&\,\,\,\langle \textit{x},\textit{r}_{n},\textit{p}_{n} \rangle, \langle \textit{x},\textit{r}_{\textit{n+1}},\textit{p}_{\textit{n+1}} \rangle) \,\vert \\&\,\,\,\forall \textit{r}_{i},\textit{p}_{i} \,.\, \textit{r}_{i} \ne \textit{r}_{\textit{i+1}} \,\wedge \, \textit{p}_{i} \ne \textit{p}_{\textit{i+1}} \, \wedge \\&\,\,\,(\langle \textit{x,is}\_\textit{a,t}_{1} \rangle, \langle \textit{x,is}\_\textit{a,y} \rangle, \langle \textit{x},\textit{r}_{i},\textit{p}_{i} \rangle) \in \\&\,\,\,(\textit{assignProperty}(\langle \textit{x,is}\_\textit{a,t}_{1} \rangle) \, \cup \\&\,\,\, \textit{alterAssignedProperty}(\langle \textit{x,is}\_\textit{a,t}_{1} \rangle)) \} \end{aligned}$$

Through experimentation, we augmented Flowchart B by using the LocatedNear (LN) relation (not shown in Fig. 2) to add a geographical context to the situation, producing ideas such as “What if a woman awoke in the sky to find she had transformed into a bird, but she could still speak?” We found that these ideas had much resonance with the premise in *The Metamorphosis*.

Additionally, it is possible to combine properties from different types; for instance, taking a lead from the surrealistic artworks of Dali, Magritte and colleagues, in flowchart C, we looked at bizarre visual juxtapositions. ConceptNet is used here to find an occupation, a vegetable, and a location related to some animal, and the flowchart produces ideas such as: “What if there was a banker underwater with a potato for a face?” Here, we are using animals and vegetables to give properties to people (described by an occupation). However, we are focusing here on controlled fictions; therefore, handling more than one type is not in the scope of this paper. This will be explored in future work.

### Exploring Scenarios

We believe that presenting a moderate amount of supporting information for an idea, such as scenarios and consequences, is conducive to encouraging the user to expand upon the idea and thus begin to own and appreciate it more. In order to generate meaningful scenarios, we need to explore how the concepts involved in the fictional idea could be affected by the transformation. For instance, the fictional idea “What if there was a little cat who was afraid of milk?” could result in negative consequences, such as the cat becoming dehydrated because of the lack of liquid, or it could affect its ability to jump from high places because its bones would be weak. It is possible as well that positive consequences emerge; for example, the cat finds more friends because it starts trying new drinks, or it invents a drink to substitute milk and becomes rich because of the idea. We could also explore reasons why the cat became afraid of milk, e.g. maybe it fears all things that are white, or it is lactose intolerant. Additionally, we could search for scenarios in which the cat tries other alternatives, or in general focus on the aspect of being afraid and think of the cat receiving therapy to overcome its fear.

Contrary to, for instance, counterfactual reasoning [34], a fictional idea may not be backed by events that precede or follow it. In many cases, those events are also fictional or they represent true facts that are not initially related to the fiction; that is, the link is fictional and is created in order to back the fiction. Thus, the procedure to find scenarios followed here consists of finding other concepts whose properties intercept with the properties of the main concept and selecting which of them are suitable to form scenarios.

There are different types of fictions, and therefore, different techniques to generate scenarios are required. Constructions 3, 4 and 5 above can be used to produce different scenarios arising from a fictional idea. For instance, from the initial fiction “What if there was a person that was half man and half bird?” we could follow with different scenarios from those constructions, for example:

- and could speak.
- but couldn’t fly.
- and could speak but couldn’t fly.

The constructions explored so far have been focused on the transference of properties from one type of concept to another, which are very common mechanisms to produce fictions. In counterfactual reasoning for example, a different mechanism is applied. Situations that are counter to the facts involve adding or removing events contradicting how things happened in the real world; furthermore, these event modifications can be seen either by the subject of the event or by the object. We have followed a similar approach here in which we alter a relation as described in construction 1, producing a fiction that either removes or reduces a property, or maximises it. Next we specify two constructions that explore different consequences arising from fictions of this type.

*Construction 6. Alternative Scenarios* If an intrinsic property of the subject is removed or reduced, other features or attributes associated with the subject could, in principle, no longer be performed. Possible scenarios are then situations in which alternatives are suggested in order to replace the property that was modified and, in this way, enable other features or attributes that depend on it. A good example of this appears in the novel *Peter Pan* of Sir James Matthew Barrie, in which the hand of *Captain Hook*, the antagonist character, is cut off and replaced with a hook.

From a transformation:

$$\langle \textit{x,r}^{\prime }\textit{,y} \rangle \in \textit{alterRelationNature}(\langle \textit{x,r,y} \rangle)$$

in which $r^{\prime }$ expresses the reduction of property *y* for subject *x*, we search for concepts *k* that represent suitable replacements for this property. This construction is controlled through the following steps:

1. The sentiment of the transformation is used to determine whether the transformed property is being reduced or removed. Thus, if:
  $$\textit{sentiment(r)} > \textit{sentiment(r}^{\prime }\textit{)}$$
  we conclude that the property expressed by the fact has been somehow weakened and therefore, an alternative scenario would be appropriate. For our work, we use the AFINN sentiment dictionary [28], which contains a list of English words, whose valency is rated through an integer value between $-$5 (negative) and $+$5 (positive).
2. Common properties of *y* are then identified to determine possible directions for the search of alternatives.
3. Concepts *k*, which have at least one property from those identified in the previous step, are selected as potential alternatives to replace *y*.
4. Concepts *k* are filtered by selecting those that are more suitable alternatives to *y* and that produce a fiction. A way of achieving this is by verifying that *y* and *k* share a number of intrinsic properties, such as shape, size, main use. However, as the information contained in KBs is limited, we use a measure of context similarity between concepts *y* and *k* in order to select appropriate alternatives. Our hypothesis is that if both concepts are used in several common contexts, then *k* may represent a suitable replacement for *y*. We further evaluate context similarity between *k* and *x* in order to make the alternative more plausible.

Formula (8) specifies the details of this construction, which returns a set of quadruplets, each of which corresponds to a different scenario:

8 $$\begin{aligned}&\textit{alternatives}(\langle \textit{x,r,y} \rangle) \\&\quad = \{(\langle \textit{x,r}^{\prime }\textit{,y} \rangle, \langle y,l,z \rangle, \langle k,l,z \rangle, \langle x,r,k \rangle) \, \vert \\&\quad \langle \textit{x,r}^{\prime }\textit{,y} \rangle \in \textit{alterRelationNature(x,r,y)} \, \wedge \\&\quad \textit{sentiment(r)} > \textit{sentiment(r}^{\prime }\textit{)} \, \wedge \\&\quad \langle y,l,z \rangle \in \textit{salient(y)} \, \wedge \, \langle k,l,z \rangle \in \textit{F} \, \wedge \\&\quad \textit{commonContext(y,k)} \, \wedge \, \langle x,r,k \rangle \notin F \} \end{aligned}$$

where *salient*(*x*) denotes a set of salient properties of *x* and *commonContext*(*y,k*) filters the alternative concepts through a measure of context similarity as it will be illustrated below.

The result from (8) is a set of quadruplets where $\langle \textit{x,r}^{\prime }\textit{,y} \rangle$ is the fiction that reduces property $\textit{y}$ of $\textit{x}$, $\langle \textit{y,l,z} \rangle$ represents a salient property of $\textit{y}$, $\langle \textit{k,l,z} \rangle$ provides a concept $\textit{k}$ that shares the same property with $\textit{y}$, and $\langle \textit{x,r,k} \rangle$ depicts a new fiction, where the initial relation $\textit{r}$ is restored through the use of alternative $\textit{k}$.

**Fig. 3:**
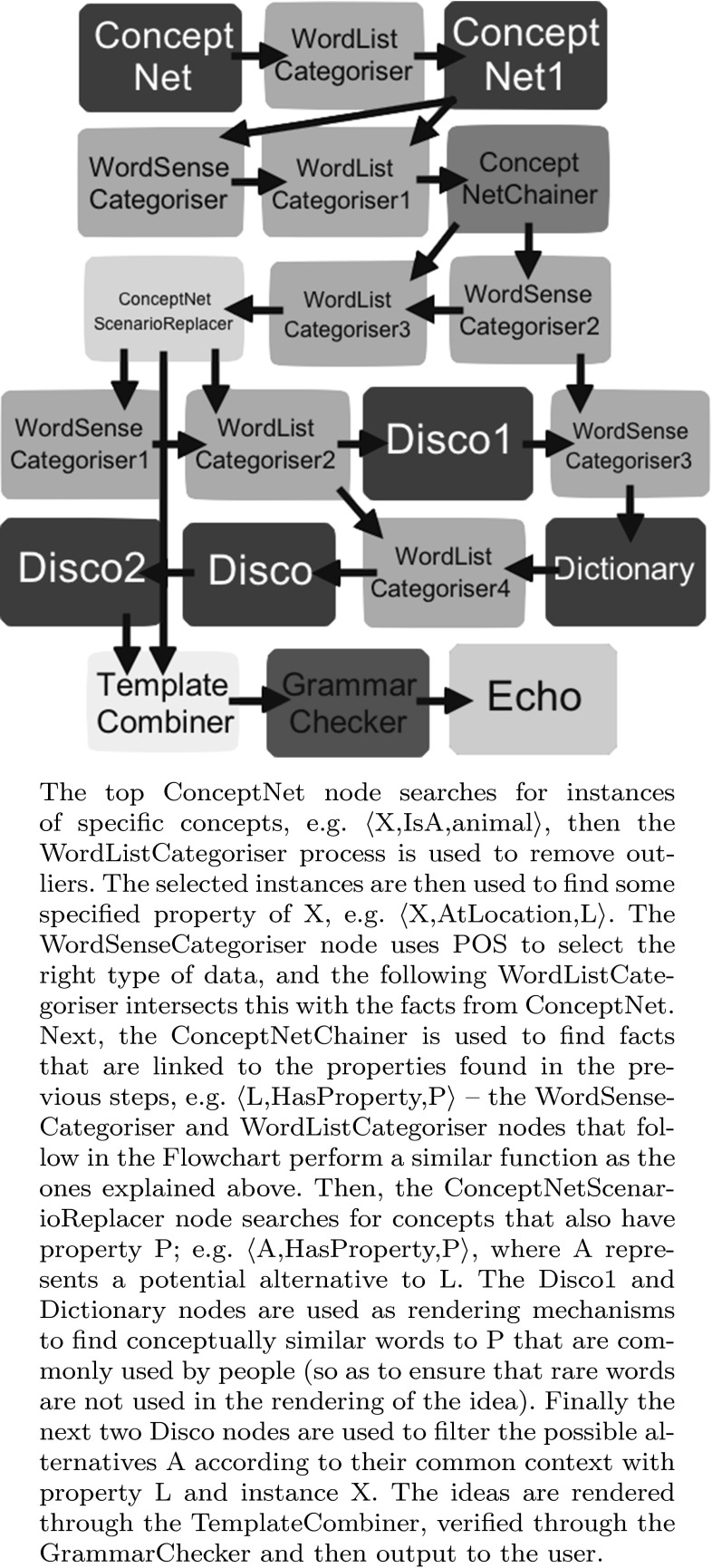
Alternative scenarios flowchart

We have experimented with this construction through the flowchart shown in Fig. 3. In order to carry out the analysis of common context, we use the semantic similarity tool Disco [15], which has an option to output common context of two input words. For instance, the following fictional idea:

What if there was an old dog, who couldn’t run any more, which he used to do for fun, so decided instead to ride a horse?

follows from the application of formula (8) to the fact $\langle \textit{dog, CapableOf, run} \rangle$:

$$\begin{aligned}&\textit{alternatives}(\langle \textit{dog, CapableOf, run} \rangle) \\&\quad = (\langle \textit{dog, NotCapableOf, run} \rangle, \\&\qquad \langle \textit{run, UsedFor, fun} \rangle, \\&\qquad \langle \textit{ride}\_\textit{horse, UsedFor, fun} \rangle, \\&\qquad \langle \textit{dog, CapableOf, ride}\_\textit{horse} \rangle) \end{aligned}$$

*Construction 7. Scope Scenarios* A different approach to finding scenarios is to explore how the scope of properties of the subject is affected when a transformation takes place. In this case, we can either: (1) find scenarios in which the scope of properties of $\textit{x}$ is enhanced because of the change, or (2) find scenarios in which the scope of properties of concept $\textit{x}$ may badly be affected. For instance, the fictional idea “What if there was a man that grew old very slowly? He could live as long as a whale” resembles the idea in Stephen King’s novel *The Green Mile* where the main character is able to live a very long life.

Again, starting from the transformation:

$$\langle \textit{x,r}'\textit{,y} \rangle \in \textit{alterRelationNature}(\langle \textit{x,r,y} \rangle)$$

we search for properties of *x* that may be affected by the transformation and then search for other concepts *k* that share the same property and can be compared in scope with *x*. Therefore, in this case, instead of focusing on properties of the object of the transformation, we focus on properties of the subject which may be affected by the transformation. This construction is controlled through the following steps:

1. Common properties *z* of *x* are identified in order to search for directions of possible scenarios.
2. Through a heuristic notion of relatedness, we determine which properties from the previous step may be affected by the transformation of property *y*. Our hypothesis is that a high score of relatedness between *z* and *y* indicates that there is a high probability that property *z* is somehow affected by the transformed property *y*.
3. For the selected properties, we then search for concepts *k* that share at least one of these properties.
4. Finally, we use the new concepts *k* to compare property *z* with the subject of the initial transformation *x*.

This is achieved as specified in (9), which returns a set of quadruplets, each of which corresponds to a different scenario:

9 $$\begin{aligned}&\textit{scopeLeft}(\langle \textit{x,r,y} \rangle) \\&\quad = \{(\langle \textit{x,r,y} \rangle, \langle x,l,z \rangle, \langle k,l,z \rangle, \langle x,as\_z\_as,k \rangle) \, \vert \\&\quad \langle x,l,z \rangle \in \textit{salient(x)} \, \wedge \, \textit{topRelatedness(z,y)} \, \wedge \\&\quad \langle k,l,z \rangle \in \textit{F} \, \wedge \, \langle x,as\_z\_as,k \rangle \notin F\} \end{aligned}$$

where *topRelatedness*(*z,y*) is used to determine whether property $\textit{z}$ can be considered as being affected by concept $\textit{y}$.

**Fig. 4:**
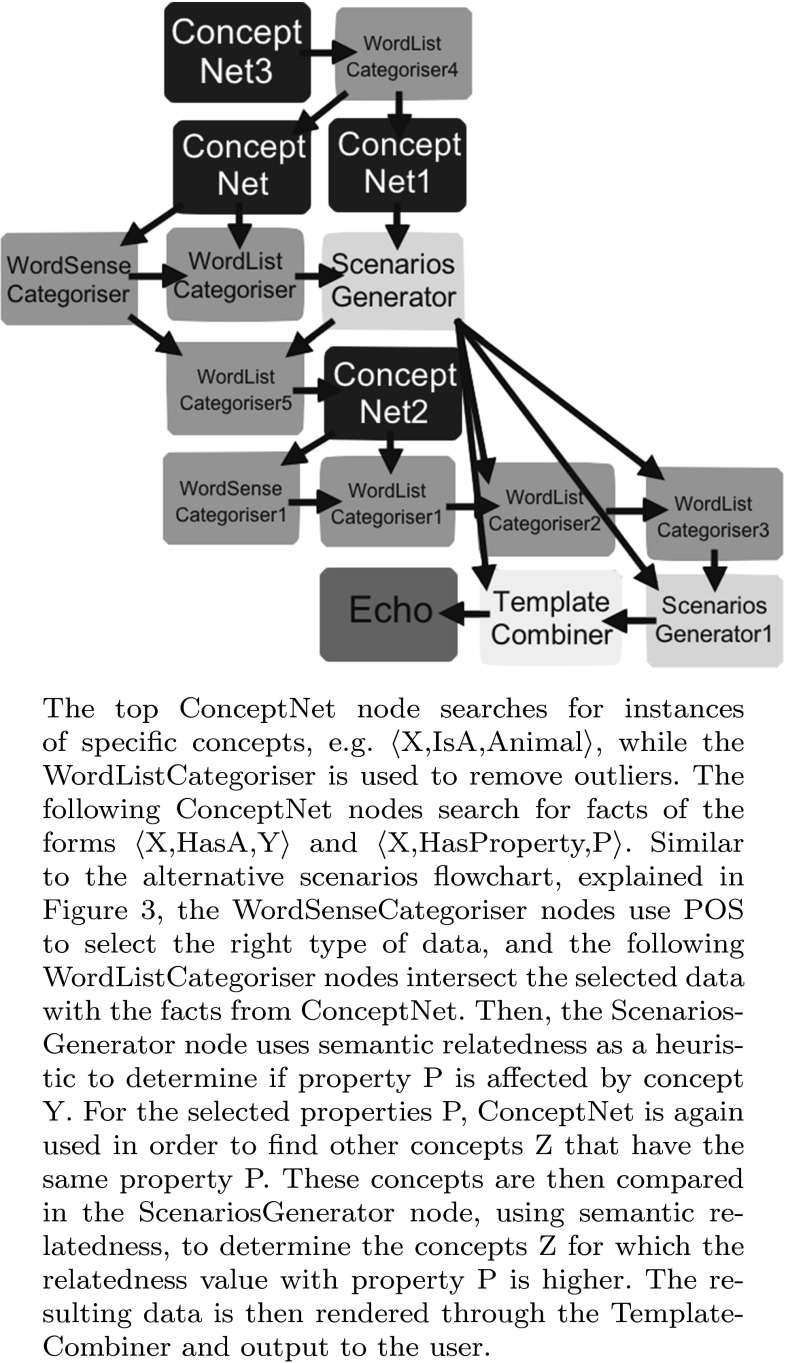
Scope scenarios flowchart

We have experimented with this construction through the flowchart shown in Fig. 4. To carry out the analysis for this construction, we require a measure of relatedness that is based on the relation instead of the conceptual meaning. That is, highly related concepts are those that are not substitutable but that are commonly used together, e.g. car and driver. This is in contrast to tools, such as Disco, that usually perform their analysis based on conceptual similarity; that is, highly related concepts are those that can replace each other, e.g. doctor and physician. We use the UMBC semantic similarity service^7^ [9], which uses *Latent Semantic Analysis* to identify words occurring in the same contexts, in order to provide a measure of relatedness.

To illustrate, take the transformation “What if there was a person that did not have an immune system?” Applying (9) first searches for the salient properties associated with the subject of the transformation, i.e. *person*. Through ConceptNet, we find that:

$$\begin{aligned} \textit{salient(person)}&= \{\textit{afraid}\_\textit{of}\_\textit{death, cruel, fragile,} \\&\quad \textit{greedy, homophobic, irrational,} \\&\quad \textit{kind, lonely, mean, sad, selfish,} \\&\quad \textit{stupid}\} \end{aligned}$$

We select the top 3 properties based on their relatedness to the object of the fiction. That is:

**Table:** 

Person		
Object of the transformation	Property of subject	Similarity score
immune_system	Fragile	0.10792253
	Mean	0.019155407
	Kind	0.017517518

Therefore, one of the possible scenarios returned is:

$$(\langle \textit{person, NotHasA, immune}\_\textit{system}\rangle, \langle \textit{person, HasProperty, fragile}\rangle, \langle \textit{eggshell, HasProperty, fragile}\rangle, \langle \textit{person, as}\_\textit{fragile}\_\textit{as, eggshell}\rangle)$$

That is, the property *fragile* of concept *person* is strongly associated with the concept *immune_system*. Then, the concept *eggshell* is also identified as having the property *fragile*. Therefore, the scenario is finally rendered as:

What if there was a person that did not have an immune system? He could be as fragile as an eggshell.

## Automated Evaluation Through Chaining

To be of value as an ideation machine, software needs to automatically identify the most valuable ideas, and investigating how best to do that will be an ongoing major challenge for the WHIM project. Part of the success of a fictional idea depends on whether the distortion of reality can be exploited to spark new ideas, to interrogate consequences and to tell stories. Given this, we developed a technique that automatically calculates the overall value of an idea by estimating an approximation to its NP.

The technique consists of building chains of relations whose starting point is the initial fact used to produce the idea, and whose following facts matched through the right-hand side concept of the previous fact and the left-hand side of the next one. This kind of reasoning is possible in most KBs due to their graph-like structure, where all nodes are connected through relations, and transitivity can be used in order to form such chains. In order to produce the chains, we selected a subset of suitable relations from ConceptNet that could be used during the construction of chains; that is, chains will only contain facts in which these relations appear. The selection of this subset was carried out through experimentation by exploring different combinations of relations and chains length. Through this study, we filtered out relations that could be subsumed by others with similar semantics, e.g. *LocatedNear*, *AtLocation* and *LocationOfAction*. We chose relations that were most frequently seen in the chains from our experiments as it would provide the best chance during the construction of chains. Furthermore, we filtered out inverse relations (negations); for instance, we use the relation *CapableOf* but not its counterpart *NotCapableOf*. This imposes further control over the semantics of the generated chains. That is, as the root of each chain is an altered fact, using the inverse relations may add unnecessary complexity to the type of short stories these chains were meant to produce. We also found that some relations did not appear very frequently in the chains, e.g. *SymbolOf*, *MemberOf*, *PartOf*. Based on these criteria, and through experimentation, we finally selected a set that covered different aspects of the ontological status of concepts, namely *IsA, CapableOf, HasA, Desires, Causes, UsedFor, HasSubevent, AtLocation, RelatedTo, HasProperty*.

**Fig. 5:**
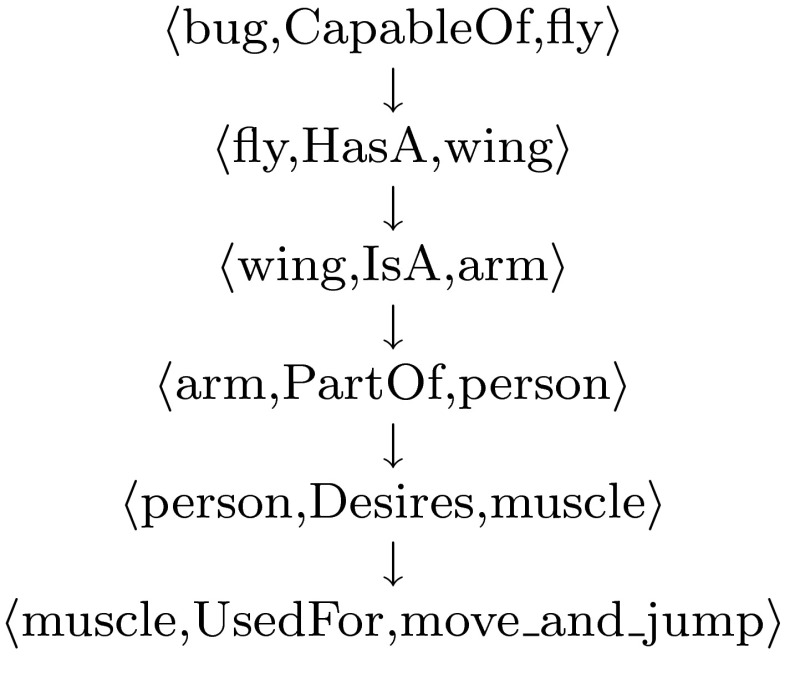
One of many possible chains of relations obtained from the fictional idea: “What if there was a little bug who couldn’t fly?”

Based on this, we can evaluate an automatically generated idea by counting the number and lengths of possible chains of facts originating from the facts at the heart of the fiction. Each chain is considered as a possible narrative that could be developed from the original idea. To illustrate this, suppose we are given the original fact $\langle$bug,CapableOf,fly$\rangle$. Then, from the seed idea *What if there was a little bug who couldn’t fly?*, the chain of relations shown in Fig. 5 can be obtained through ConceptNet.

One possible interpretation of the chain of facts in Fig. 5 is:

There is a little bug who can’t fly, as he has arms instead of wings. He would develop arm muscles to move and jump instead of flying.

Through this interpretation, we could possibly imagine a Disney film about a little bug who, even though he cannot fly, overcomes adversity with superstrength because of his muscular arms.

Automatically generating such interpretations is very much future work. However, such chains could still be of use. In particular, our hypothesis is that—while each chain might be rather poor and difficult to interpret as a narrative—the volume of such chains can indicate the potential of the idea. Hence, our evaluation method gives ideas with more chains associated with them, a higher score than those with fewer chains.

We implemented this technique to take a given idea and develop chains up to a specified length with no loops or repetitions. Hence, facts with many chains are ranked higher than chains with fewer, and longer rather than shorter chains will also push a fact up the rankings. Regarding ConceptNet, often there are no chains for a fact, and if there are, the number depends on the nature of the objects being related and the relation. For instance, looking at the transformations applied in Fig. 1, where we applied construction 1 to different properties of animals, we found these percentages of facts had non-trivial chains:

**Table:** 

CapableOf	Desires	HasA	HasProperty	IsA	LocatedNear
20	50	63	28	48	100

## Curation Analyses

Colton and Wiggins [3] introduce the term *curation coefficient* as an informal reading of the *typicality*, *novelty* and *quality* measures put forward in [33]. In essence, this involves a project team member examining the output from their generative software, and calculating the proportion that they would be happy to present to others. This form of assessment is being embraced in computational creativity research as a way to measure progress during the development of CC systems. Based on this, an initial estimation of the value of the automatically generated output can be drawn, as well as a baseline assessment that can be used in order to compare with future versions of a system. As the analysis is performed by the authors themselves, there is some subjectivity in it; however, the appreciation of what-ifs is in itself subjective to each individual tastes and beliefs. Having this into account, in the next section we extend the evaluation of fictional ideas through a crowd-sourcing experiment that involves 135 participants. This provides a broader and approximate objective estimation of the value of the ideas that are currently produced by the system, since it reflects the preferences of a crowd rather than a few individuals.

For our purposes here, the curation analysis is performed based on slightly lower criteria than that described in [3]: we took all the ideas from each method, or a sample when there were too many, and recorded how many were suitable for assessment, i.e. the proportion of ideas that were both understandable and fictional, without any judgement of quality. This value is called *the curation coefficient*.

In Fig. 2, we present flowcharts A to D for generating fictional ideas using ConceptNet. Facts in ConceptNet are scored for truth likelihood, and flowchart A is parameterised by a threshold, $T_1$, for the minimum score that ConceptNet facts must achieve to be used. Flowchart B uses ConceptNet twice and hence has thresholds $T_1$ and $T_2$. Flowcharts C and D were not parametrised and used a fixed ConceptNet threshold of 1. Table 1 shows the number of ideas (yield) that each flowchart (FC) produced, with various threshold settings. The table also shows the curation coefficient (C-Coeff), i.e. the proportion of understandable and (largely) fictional ideas. We see that the yield reduces as higher thresholds $T_1$ and $T_2$ are imposed, but the C-Coeff increases, because fewer spurious or nonsensical facts are inverted for the ideas. In one case for flowchart B, by setting $T_1$ and $T_2$ to 5, we were able to produce a set of 27 ideas with a 100 % C-Coeff. We noted an average yield of 190.4 and an average C-Coeff of 84.1 %, which we find encouraging.

**Table 1:** Curation analysis: constructions 2–5

FC	Example	$T_1$	$T_2$	Yield	C-Coeff (%)
A	He was half man, half bird	1	–	97	72
		3	–	21	90
		5	–	14	93
B	He was half man, half fish, who could live in a lake	5	1	453	78
		5	2	94	88
		5	5	27	100
B	He was a cat, but he could still write	5	1	48	88
		5	3	7	100
C	Composer in a nest with turnip for a face	–	–	272	56
D	Dolphin that is made out of gold	–	–	871	76
	Average			190.4	84.1

In Table 2, we show the curation analysis for the scenarios flowcharts. In the case of the alternative scenarios, we explored three ConceptNet relations, which express the initial transformation and for which the alternative is searched. Examples of the ideas generated by each flowchart are:

- *AtLocation* What if there was an old book that couldn’t find a study that was quiet? But instead, he found a special style of hush that was so cheerful that the old book didn’t want the quiet study anymore.
- *CapableOf* What if there was a poor pen that couldn’t write because he didn’t have creativity? So he decided to pretend instead.
- *HasA* What if there was an ox who lost his horn and couldn’t communicate? But then he discovered that a call would solve his problem, so he forgot all about his old horn.

In the case of the scope scenarios, we use as the initial relation *HasA* and explored different topics by changing the type of the subject of the idea. Examples of the ideas generated by each flowchart are:

- *Animals* What if there was a clumsy sheep who lost her farm and then suddenly became as domestic as a dog?
- *Machines* What if there was a fan that, even though it didn’t have a motor, was still as noisy as a thunder?
- *Objects* What if there was a bridge whose substructure ran away but was still as sturdy as a house?
- *Occupations* What if there was a clumsy guard who lost her uniform and then suddenly became as right as a claim?
- *Things* What if there was a tree that, even though it didn’t have a branch, was still as alive as a cat?

One threshold is used in Table 2, $T_1$, which refers to the ConceptNet likelihood score assigned to each ConceptNet process used in both flowcharts. In this case, we have used a fixed threshold of 1. As can be observed, the C-Coeff in both flowcharts are lower than those in Table 1. We believe this is because although fictional, the success of these flowcharts depends on establishing a credible relationship between the concepts involved in the what-if. This is not strongly required in contexts such as surrealist art or stories like the metamorphosis, which are the type of fictions explored in Table 1. To illustrate this point, we use some of the ideas rejected through the curation analysis. For instance, the concepts involved in the following idea are completely unrelated, making it too difficult to interpret: “What if there was an old projectile that couldn’t find a tornado that was dangerous? But instead, he found a special style of rattlesnake that was so risky that the old projectile didn’t want the dangerous tornado anymore”. A similar case occurs with the idea: “What if there was an old person, who couldn’t feel anymore, which he used to do to break, so decided instead to smash?” In this case, it is difficult to establish a relation between the concepts involved, making the idea difficult to understand. Additionally, some of the ideas failed on their level of fictionality; for instance, the idea: “What if there was a bicycle that, even though it didn’t have a brake, was still as expensive as jewellery?” can be easily thought as plausible, maybe one can think of a collectable bicycle. Likewise, the idea: “What if there was a clumsy machine who lost her part and then suddenly became as mechanical as a motor?” is not fictional since losing a part does not stopped a machine from being mechanical.

**Table 2:** Curation analysis: constructions 6 and 7

FC	Topic	$T_1$	Yield	C-Coeff (%)
Alternatives	AtLocation	1	15098	33
	CapableOf	1	23	28
	HasA	1	116	14
	Average		5079	25
Scope	Animals	1	82	25
	Machines	1	386	52.5
	Objects	1	857	15
	Occupations	1	51	17.75
	Things	1	522	27
	Average		379.6	27.45

However, the curation analysis for the alternatives scenarios flowchart was collected ignoring the common context heuristic explained in construction 6. This is with the aim of evaluating the effectiveness of this heuristic. We imposed this heuristic to the ideas generated through the *AtLocation* relation and found that the C-Coeff increased from 33 to 51 %, which is encouraging. We plan to further experiment with relatedness and common context heuristics.

Through our approach, data mined notions of reality were altered, respectively; hence, the ideas were largely fictional. With respect to nonsensical ideas, we learned that control over quality could be exerted, at the expense of yield, through the usage of the ConceptNet thresholds and common contexts measures.

## A Crowd-Sourcing Evaluation

Ultimately, the fictional ideas we want to automatically produce will be for general consumption. Hence, a large part of the WHIM project will involve crowd-sourcing responses to fictional ideas and using machine learning techniques to derive an audience model that can predict whether generated ideas are going to be of value. To get a first tranche of feedback from the general public, we focused on ConceptNet ideas within the context of anthropomorphised animal characters which could feasibly appear in a Disney animated film. This context was chosen because Disney movies are familiar to most people and somewhat formulaic; hence, we could be reasonably confident that when we surveyed people, our questions would be interpreted appropriately.

During a pilot study reported in [19], we focused on ideas generated by the CO relation in the second ConceptNet node of flowchart B in Fig. 2; that is, we studied ideas of the type: “What if there was a little X, who couldn’t Y?” With an online survey of four questions, we asked 10 English-speaking participants to rank the same list of 15 such Disney characters, in terms of (a) general impression (GI) (b) emotional response (ER) provoked (c) *narrative potential*: number and quality of potential plot lines imaginable for the character, and (d) how surprising they found the character to be. Our aim was to measure the influence of emotional provocation, NP and surprise on GI. Recall that we wrote routines to produce chains of ConceptNet facts. The 15 Disney characters in the survey comprised 5 from ideas with no chains, 5 from ideas with multiple chains, and 5 ideas where the RHS of a ConceptNet fact was replaced with a randomly chosen verb.

This pilot study showed that ConceptNet ideas were ranked much higher than the random ones for three questions, with average ranks of 5.21 versus 10.98 for GI, 6.08 versus 11.5 for emotional provocation and 5.00 versus 11.32 for potential for NP. Within the ConceptNet examples, those with chains were ranked slightly higher than those without: average ranks of 4.78 versus 5.21 for GI, 3.42 versus 6.08 for ER and 4.68 versus 5.00 for NP. However, when assessing levels of surprise, the random ideas were ranked as best with an average rank of 4.48 versus 8.18 for ConceptNet ideas with no chains, and 8.44 for those with chains. On reflection, we determined that this resulted from an inconsistent interpretation of the word “surprising”. We also found in the pilot study that there was a strong positive correlation *r* between GI and both ER ($r=0.81$) and NP ($r=0.87$), confirming that both these elements are key components of participants’ GIs of value. However, we found a strong negative correlation between GI and surprise ($r=-0.77$). Hence, this suggests that more surprising ideas aren’t generally well received.

Building on and learning from the pilot study, we undertook a larger-scale experiment. For this, we used three sets of Disney characters generated using ConceptNet facts with the CO relation as before, in addition to the Desires (D) relation (“What if there was a little X who was afraid of Y?”) and the LN relation (“What if there was a little X who couldn’t find the Y?”). In order to evaluate participants’ preferences, we designed four surveys: one per relation, and a fourth that mixed Disney characters from the three relations. In order to prevent bias or fatigue, each participant completed only one of the surveys.

Each survey consisted of four questions that asked participants to rank Disney characters in order of their GI of the character’s viability, the degree of ER they felt upon reading and interpreting the idea of the character, the quantity and quality of the plot lines, i.e. NP, that they felt might be written about each, and to what level each character met their expectation (LE) of a Disney character. This last question replaced the final question from the pilot study. The relation-focused surveys had a set of 14 ideas, eight ConceptNet non-chaining (NC) ideas (i.e. only one associated chain) and six ConceptNet chained (CC) ideas (i.e. with multiple associated chains)—random ideas were not evaluated as they scored significantly worse in the pilot study. The mixed survey used a set of 15 CC ideas, five per relation. These ideas were chosen by sampling systematically at equal intervals in terms of chaining score.

**Fig. 6:**
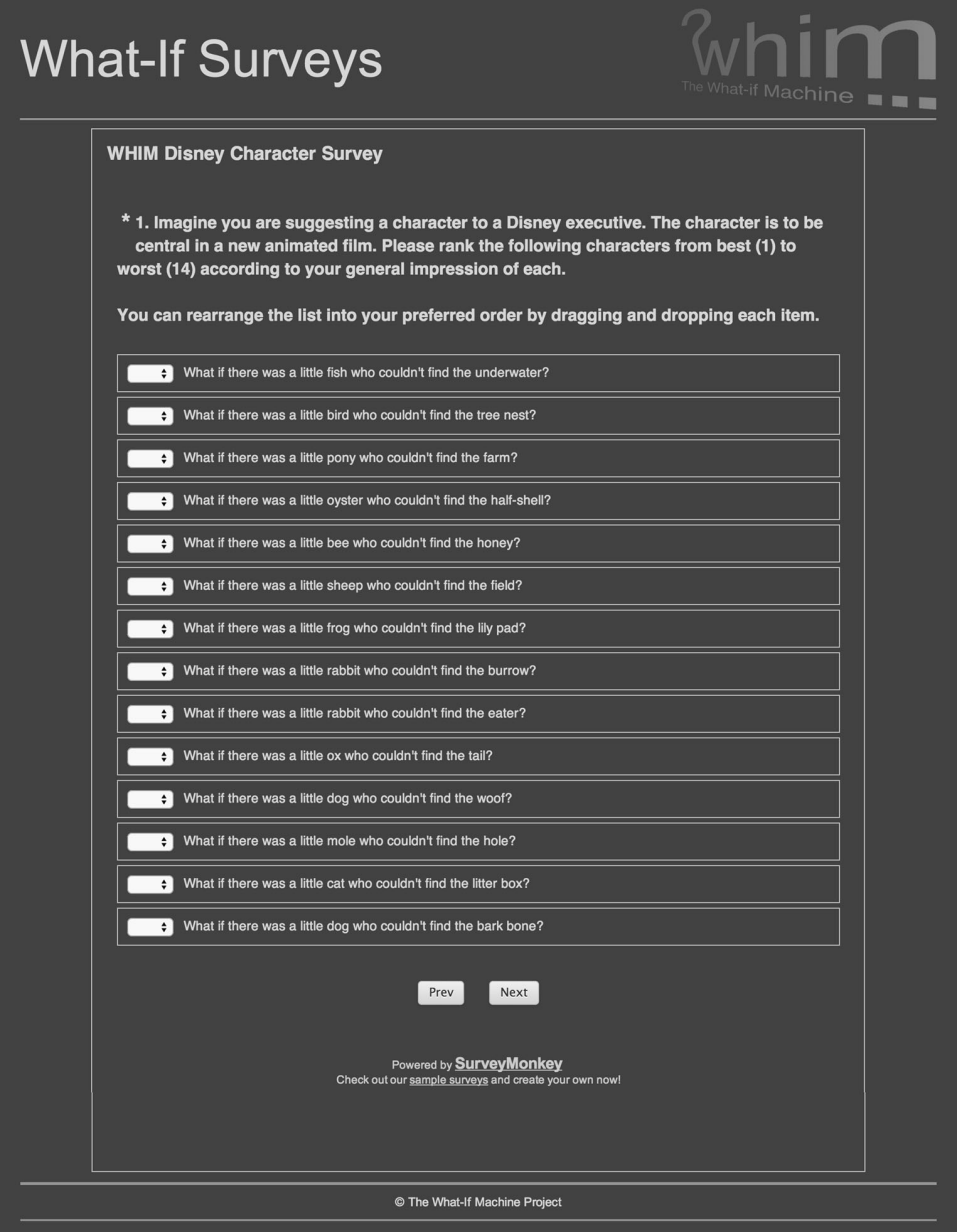
Example survey carried out in the crowd-sourcing experiment

The crowd-sourcing experiment was conducted using the SurveyMonkey system. This platform was chosen because it was simple to use—from the view of designing the surveys as well as taking part in them—it provided us with functionalities to personalise the questionnaires as well as diverse statistics from the data that could be used in the post-analysis. A screenshot of one of the questionnaires used is shown in Fig. 6. Contrary to the pilot study, the crowd-sourcing evaluation was not restricted to native English speakers. Because the what-ifs produced at the moment are meant for public consumption, there was no specific background or restrictions imposed to participants, most of which were friends or academics from the departments of the partners institutions involved in the WHIM project. Moreover, in order to control the results, as we mentioned before, we limited each survey to 15 ideas only in order to avoid fatigue. Furthermore, we only selected answers from those participants that completed all the four questions and remove also those whose time of completion was too short or whose level of confidence was low—this was asked as part of the survey; details about this are provided in the following section.

### Results

A total of 135 participants completed the crowd-sourcing experiment, with at least 27 participants per survey. We had respondents with different levels of fluency: 1 was at a basic level, 12 consider themselves at an intermediate level, 68 participants were fluent, and 54 were native English speakers. These figures show that at least 90 % of the participants were fluent or native, which provides a high level of confidence in the reliability of the results. Moreover, 64 participants were female, 70 were male, and 1 person preferred not to specify their gender. This shows an almost even participation from both genders. The participants were between 18 and 74 years old; more specifically, 12 were in the age range between 18 and 24 years old, 74 in the range 25–34, 33 in the range 35–44, 7 in the range 45–54, 7 in the range 55–64 and 2 in the range 65–74. The highest concentration is seen in participants between 25 and 34 years old; however, most age ranges were represented in the surveys. After completing the surveys, we asked the participants to select their level of confidence, between *very low, low, medium, high* and *very high*, when answering each question. Table 3 shows that most of the participants answered each question with a medium level of confidence or higher. This increases the confidence we have in the results.

**Table 3:** Percentage of participants who answered each question with a medium level of confidence or higher

	Percentage of participants			
Question	CO	D	LN	Mixed
GI	97	90	94	96
ER	97	90	88.5	92.5
NP	78	82.5	83	85
LE	85	80	80	78

Table 4(a) shows the average rankings given for each class of ideas in the relation-focused surveys. As suggested in the pilot study, in general, the CC ideas are ranked around 1 position higher than the NC ideas. This supports the hypothesis that the CC evaluation technique provides a reliable measure of value for fictional ideation using ConceptNet. Using a Friedman test comparing the mean ranks for CC and NC ideas in each response, we found that the difference between their ranks is highly significant overall ($p<0.001$). This effect remained significant across all question and survey subgroups.

**Table 4:** Crowd-sourcing experiment results for four surveys: CO, D, LN and Mixed

(a) Average participant rankings for three relation-focused surveys by type of idea: non-chaining (NC) and ConceptNet chaining (CC)															
Q	CO		D		LN		Avg								
	NC	CC	NC	CC	NC	CC	NC	CC							
GI	7.41	7.62	7.76	7.15	8.05	6.77	7.74	7.18							
ER	7.88	7.00	8.03	6.80	7.85	7.03	7.92	6.94							
NP	7.85	7.04	8.03	6.80	7.95	6.90	7.94	6.91							
LE	7.95	6.90	8.15	6.63	8.01	6.81	8.04	6.78							

(b) Average participant rankings for mixed survey by inverted relation															
Q	Mixed														
	CO	D	LN												
GI	7.48	7.70	8.81												
ER	6.55	8.44	9.01												
NP	7.86	7.48	8.66												
LE	7.24	8.46	8.30												

(c) Average rank correlation between all the questions of the four surveys: general impression (GI), emotional response (ER), narrative potential (NP) and level of expectation (LE)															
	GI&ER	GI&NP	GI&LE	ER&NP	ER&LE	NP&LE									
Avg. Corr. ($\tau$)	0.34	0.36	0.31	0.35	0.32	0.37									

(d) Rank correlation between av. participant rankings and chaining rankings															
Q	Correlation ($\tau$)														
	CO	D	LN	Mixed	Avg										
GI	0.09	0.25	0.27	−0.24	0.09										
ER	0.17	0.25	0.26	0.26	0.23										
NP	0.22	0.22	0.21	0.23	0.22										
LE	0.14	0.27	0.22	0.08	0.17										

(e) Rank correlation between average participant rankings and ConceptNet relations rankings															
Q	Correlation ($\tau$)														
	CapableOf			Desires			LocatedNear			Mixed			Avg		
	IsA	CO	CB	IsA	D	CB	IsA	LN	CB	IsA	Rel	CB	IsA	Rel	CB
GI	0.25	0.19	0.31	0.42	0.17	0.40	−0.17	0.34	−0.17	0.20	0.27	0.31	0.17	0.24	0.21
ER	0.18	0.22	0.25	0.51	0.10	0.49	−0.07	0.21	−0.03	0.22	0.40	0.39	0.21	0.23	0.27
NP	−0.02	0.07	0.03	0.46	0.07	0.44	−0.07	0.27	−0.03	0.23	0.26	0.26	0.15	0.16	0.17
LE	0.39	0.11	0.44	0.46	0.10	0.44	0.02	0.17	0.06	0.18	0.29	0.31	0.26	0.16	0.31

Table 4(b), which presents the results from the fourth survey, shows that, in general, the CO ideas were ranked highest, followed by the D ideas and then the LN ideas. A Friedman test showed these differences to be highly significant overall ($p=0.001$). Our interpretation is that participants considered that, in some cases, the D ideas and LN ideas failed with respect to the feasibility of the fictional characters they portrayed; therefore, they were ranked lower. More specifically, respondents suggested that they felt apathy towards anthropomorphisations such as “a little goat who is afraid of eating” (D idea), which threatened fundamental aspects of animals’ lives, as well as ideas such as “a little oyster who couldn’t find the half shell” (LN idea), which were found difficult to interpret. On the contrary, participants pointed out that some of the CO ideas were “reminiscent of existing cartoons”, placing them into a higher rank, e.g. “a little bird who couldn’t learn to fly” (which resembles the plot of the animated film *Rio*). These type of participant judgements played an important role when ranking the ideas, resulting in a clear overall preference for the CO ideas.

We also wanted to confirm the pilot study suggestion that ER, NP and LE are key components of participants’ GI of value. We used a Kendall rank correlation coefficient ($\tau$) for this analysis. Table 4(c) shows the average correlation results between all the components, showing a positive correlation between all the surveyed components. However, a Friedman rank-sum test indicated that the particular differences between correlation values are not significant ($p=0.2438$); that is, all question pairs were similarly correlated.

Table 4(d) shows the correlation between the chaining scores and the overall rankings of the participants. We see that weak positive correlations were found for most of the aspects evaluated in the four surveys and the chaining scores. These results confirm that, as suggested in the pilot study, the chaining technique can be used as a measure to evaluate fictional ideas, and we plan to investigate the value of generating other semantic chains to increase the effectiveness of this technique. Table 4(d) also shows that a weak negative correlation exists between participants’ GI and the chaining scores for the mixed survey. This suggests that participants found it more difficult to decide on the rankings when the rendering of the ideas was mixed.

Finally, two facts are used for each idea generated with ConceptNet: facts that tagged words as animals with the *IsA* relation, and facts to be inverted, which use the CO, D and LN relations. Table 4(e) shows the results of calculating the correlation between the average participants’ rankings and each ConceptNet fact score, as well as the combination of both (CB). We see that, except for the LN survey, most of the results show a weak positive correlation. This supports the finding from the pilot study that the values people project onto ideas are somewhat in line with the score assigned by ConceptNet to the underlying facts. Moreover, the highest correlations are presented in the D survey with the *IsA* relation. We believe that people tend to rank higher ideas associated with more common animals, such as dogs or cats, used in multiple ideas of the D survey, than ideas involving relatively uncommon animals, such as ponies, moles or oxen, which were used in the LN survey.

The correlations between the participants’ rankings and the chaining and ConceptNet scores [(Tables 4(d) and 4(e)] led us to believe that these scores could be used to predict people’s preferences when ranking fictional ideas. To test this hypothesis, we used the Weka machine learning framework [8]. We provided Weka with the scores of: CC, ConceptNet strength for the IsA relation, ConceptNet strength for the inverted relations, word frequencies for the LHS and RHS of inverted facts, and semantic similarity between the LHS and RHS of inverted facts, obtained using the DISCO system.^8^ We classified each idea into *good* (top 5), *bad* (bottom 5) or *medium* (middle 5) based on the average participants’ rankings. We tested a variety of decision tree, rule-based and other learning mechanisms, with the results given in Table 5, along with the name of the learning method which produced the best classifier. We found that the RandomTrees approach consistently performed well, but was only the best method for two aspects of evaluation. We used Weka to perform a paired *t* test, which showed that the predictors are significantly better than the majority class classifier (MCC)—which simply assigns the largest class as a prediction—with up to 95 % confidence.

**Table 5:** Predictive accuracy for GI, ER, NP and LE

	MCC	GI	ER	NP	LE
Method	ZeroR	Ridor	RandTree	NBTree	RandTree
Accuracy (%)	35.08	49.12	56.14	43.85	54.38

MCC value was the same for all evaluated aspects, i.e. GI, ER, NP and LE

## Conclusions and Future Work

While essential to the simulation of creative behaviour in software, fictional ideation has barely been studied in computational creativity research. We have implemented an approach to automated fictional ideation based on the manipulation of facts from KBs. We presented a baseline methodology for assessment, in the form of a curation analysis and a crowd-sourcing study where participants ranked fictional ideas. The curation analysis showed that when guided in a strong context such as Disney characterisations, automated ideation methods work well, but they degrade when the context becomes weaker. The crowd-sourcing study showed that an inference chaining technique—inspired by the hypothesis that ideas can be evaluated through narratives involving them—provides a reliable measure of value with which to assess the quality of fictional ideas. Also, we found positive correlations between the rankings of GI and each of ER, NP and LE, showing that these are key elements of participants’ GI of fictional ideas. Finally, we demonstrated that machine learning techniques can be used to predict how people react to a fictional idea along these axes, albeit only around 50 % predictive accuracy.

The generation and assessment of narratives will be a key factor, enabling the system to curate its output. We will derive a theory of idea-centric narratives and implement methods for generating and assessing ideas in terms of the quality/quantity of narratives they appear in. We believe that our CC technique shows much promise as supported by the positive correlations found between our results and the rankings from the crowd. Although a person may be able to take an idea with a trivial chain and make it a very exciting story, imagination, a property inherent to human cognition, plays an important role in such cases. Some studies have been carried out in order to understand how appreciation of an artefact changes with time, interaction, cultural and social beliefs, etc. [12, 23, 35]. In [12], for instance, it is suggested that creativity is associated with the positive response from audiences towards artefacts produced, regardless of the author, and that are other factors people are influenced by (such as culture) that determine the value of an artefact. These studies however have focused on creativity at the human level, whereas here, and in general in computational creativity, we are interested in creativity at the level of computer systems. Douglas Hofstadter has highlighted this important difference in his research on the Copycat system:

Real cognition of course occurs in the essentially boundless real world, not in a tiny artificial world. [11, p. 105]

That is, although we take human appreciation into account by modelling their preferences through crowd-sourcing studies, to model aspects of cognition that affect appreciation such as culture, social beliefs is out of the scope of this work. Ultimately, we are taking an engineering approach (rather than a cognitive approach) to fictional ideation and our aim is to build a working computational system able to generate textual what-if ideas as a study in computational creativity. As such, we will explore computational approaches such as [14, 37] which explore the evaluation of creative systems and their outputs.

The C-Coeff associated with the scenarios constructions was around 25 %, which means that 1 in 4 of the fictional ideas was assessable. At this stage in the project, this is an encouraging result. We plan to use open information extraction techniques for Web mining, which we hope would increase the yield and quality of the generated ideas. Moreover, some scenarios may be more possible than others, and this may affect how the idea is perceived. Scenarios with a high probability of occurrence may not be that interesting, since there is not much surprise in them happening; however, scenarios with zero probability may be at too low threshold, making the idea completely infeasible. Contextual semantic similarity tools have helped us evaluate scenarios; however, these tools are previously trained on general properties. We are going to explore the possibility of training new word vector representations through the Word2Vec system [25] so that they are tailored to our ideation process.

The WHIM project is primarily an engineering effort to build the What-if Machine as a Web service and interactive engine, which generates fictional ideas and provides motivations and consequences for each idea, potential narratives involving it, and related renderings such as poems, jokes, neologisms and short stories. The first version of the What-if Machine is available online.^9^ Users can parameterise the method for exploration, or simply click the “I’m feeling lucky” button. The online system uses some of the flowcharts presented in this paper and the Flux Capacitor [39], to produce fictional ideas. The declarative definitions presented here describe the process followed by the flowcharts. Where a step or feature of the declarative definition has not been explicitly programmed into them, they have been semantically followed in their construction; for instance, hand-picked selection of inversions of ConceptNet relations, e.g. *desires* becomes *afraid of*. The automation of all the features expressed in the declarative forms is a constant effort made by the WHIM consortium in order to produce a more general system. Finally, the online system will collect constant feedback from the general public about the quality of its ideas; therefore, new and improved models will be produced between some intervals of time. We also hope this implementation would help promote fictional ideation as a major new area for computational creativity research.

## Footnotes

1 www.thepaintingfool.com.

2 www.whim-project.eu.

3 conceptnet5.media.mit.edu.

4 www.whim-project.eu/whatifmachine.

5 From the point of view of the authors.

6 http://swoogle.umbc.edu/SimService/GetSimilarity.

7 swoogle.umbc.edu/SimService/index.html.

8 www.linguatools.de/disco/disco_en.html.

9 www.whim-project.eu/whatifmachine.
